# The RNA World: 4,000,000,050 years old

**DOI:** 10.3390/life5041583

**Published:** 2015-10-22

**Authors:** Niles Lehman

**Affiliations:** Department of Chemistry, Portland State University, PO Box 751, Portland OR 97207, USA; niles@pdx.edu; Tel.: +1-503-725-8769

The RNA World is now some four billion years behind us, but only recently turned 50 as a human hypothesis. As early as 1962 Alex Rich suggested that RNA might have a phenotype in addition to its informational role [1]. In the Special Issue of *Life* devoted to The Origins and Early Evolution of RNA, 17 papers explore a remarkably broad range of topics surrounding this hypothesis. I would not go so far as to say the hypothesis is experiencing a mid-life crisis. However it is clear that it has generated a spectrum of viewpoints, from ardent devotees to outright skeptics. I perceive all these vantage points as giving us a richer appreciation of the chemical origins of life.

The Issue was initiated by a cogent argument from Scott and colleagues that the 2´,3´-cyclic phosphate version of nucleotides should be considered a viable prebiotic source of monomers for the abiotic polymerization into RNA [2]. Their argument has not only a thermodynamic basis, but draws support from the fact that such nucleotides have now a plausible prebiotic synthetic route and from the observation that many self-cleaving ribozymes (*i.e*., RNA enzymes, and thus operating on reversible reactions) generate such products. Martin *et al.* also briefly consider the role of 2´,3´-cyclic phosphates in their paper, which is an in-depth survey of the problems and potentials of RNA ligase, nucleotide synthase, and RNA replicase ribozymes [3].

The key problem of how RNA nucleotides, if present prebiotically, could polymerize into oligomers in the face of an uphill battle against hydrolysis in an aqueous medium, was taken up by two papers in the Special Issue. Hashizume [4] details the pH and ionic conditions that affect adsorption of nucleotide components – nucleobases, ribose, and phosphates – onto clay, which has often been proposed as a surface catalyst for abiotic RNA polymerization. And Pino *et al.* revisit the role of formamide as both a reactant and a solvent for activated nucleotide synthesis that obviates much of the water problem [5]. Of note is that their reaction schemes in formamide also have the potential to generate 2´,3´-cyclic phosphates. Moreover they highlight the role of the 3´-OH of RNA oligomers to participate in recombination chemistry, a powerful way to rapidly promote oligomer diversity, as our group has also noted [6,7].

The transition from “short-mers” to “longer-mers” was also investigated by Mungi and Rajamani [8] and Kanai [9]. The former provided an empirical study that invoked both dehydration-rehydration cycles and lipid assistance to define optimal conditions for abiotic polymerization. Here it was shown that nucleobases hydrolysis to create abasic residues in an oligomer was a common process and that there may have been evolutionary pressure to minimize this event. The latter examined tRNA structures from organisms in which these RNAs are “split” and located in disparate locations in the genome, and concludes that longer RNAs such as tRNAs could result from the annealing and subsequent reaction, likely recombination, of the two (or more) pieces.

Once RNAs were long enough, the RNA World hypothesis generally turns to how the evolutionary process could speed up. Kun and Szathmáry, utilizing a recent influx of experimental data on RNA function, explore the nature of RNA fitness landscapes [10]. A very important outcome of their analysis is that there should be four general types of RNA structure: critical, connecting, neutral, and forbidden. Each can be obtained in multiple ways, and the epigenetic interactions among them are crucial in the progress of RNA molecules through sequence space during evolution. From another perspective, Witzany likens RNA motifs to agents in a molecular society [11]. This viewpoint allows us to employ cooperative interactions among molecules and group dynamics to understand evolutionary change.

Another “next step” in RNA World discussions is the origin and evolution of the genetic code. This problem was pondered long ago by Jukes [12], Woese [13], and others, and has spawned a tremendous amount of speculation, perhaps too much [14]. Nevertheless it is indeed a fascinating – and important – question. Code evolution theories tend to fall into two broad groups, with some overlap. The first group argues code evolution from symmetry, asymmetry, and mathematical points of view. José *et al*. [15] take this approach by applying group theory (common to inorganic chemists but less so to biologists) to the code. They describe “Genetic Hotels” as a way to visualize the genetic code such that fingerprints of past evolutionary events can be detected. The second group uses chemical relationships between and among RNA and amino acid residues to reconstruct code history. Fontecilla-Camps [16] takes this approach and makes the intriguing point that non-proteinaceous amino acids (by today’s accounting) may actually have been some of the first components of primitive peptides, and that the modern code derived in successive stages as newer amino acids replaced older ones. Beier *et al*. [17] also take a physico-chemical view of the evolution of the genetic code. They take the novel tact of examining a broad range of protein-RNA interactions in extant biology to deduce what determined which amino acids were paired with which codons. To bring everything together, Hartman and Smith [18] also consider non-standard amino acids as being early members of the code. They describe a logical scenario that starts with codons only comprised of G and C, with the rest being non-coding *e.g*., [19], and working all the way up to the composition of the ribosome and its tRNA complement.

Once the RNA World had a foothold, a next logical question is, how did life bring in DNA? In today’s biology, DNA synthesis requires a very challenging enzymatic reaction catalyzed by the ribonucleotide reductase (RNR) protein enzymes, which use RNA as a substrate. Given the complexity, yet apparent antiquity, of this reaction, the evolutionary provenance of these enzymes is of extreme importance. Lundin *et al*. [20] provide a very comprehensive review of RNRs and consider how the three contemporary classes of these proteins may have come to be from a more primordial type(s). This review forces us to consider the key dilemma of how radial (in the electronic sense) chemistry originated, because it is not yet clear how – or if – ribozymes could perform this type of reaction.

The last group of papers in the Issue deal with the highly probable case where the RNA World was not strictly consisting of RNA, and that peptides or other molecules either initiated life or co-evolved with RNA. The so-called “RNP World” – for ribonuleoprotein world – posits that RNA could not have had enough catalytic prowess without the help of peptide cofactors. This is a rather attractive hypothesis and various authors have varying degrees of opinions on its strength. The “Strong RNP World” (by analogy to the “Strong RNA World” hypothesis [21]) is advocated by Carter [22], who points to a partnership between oligonucleotides and oligopeptides as an obvious means to solve the problem of simultaneous coding and catalytic function by a single biopolymer (*i.e*., RNA *per se*). The same stance is taken by van der Gulik and Speijer, who argue forcefully on catalytic grounds that an RNA world without amino acids could never have existed [23]. Smith *et al*. [24], perhaps to the first prebiotic chemists to derive inspiration from the singer Joni Mitchell, remind us that even proteins can store information and direct the synthesis of other proteins (as evidenced by prions and amyloids). This observation is often overlooked, and provides cogent rationale for the argument that nucleic acids and polypeptides may have been in a mutualistic evolutionary relationship from the very start. Finally, Wächtershäuser [25] makes perhaps the most forceful argument against the Strong RNA World point of view by tracing the evolutionary events back to simple, surface-mediated autotrophic reactions. He reiterates that these reactions were driven by metal catalysts, and that until a robust set of metabolic feedback and feedforward cycles were established, there would be no logic in discussing the advent and evolution of informational polymers. Wächtershäuser’s theory is that these polymers co-evolved after metal catalysis became robust, and concludes his paper with the following quote, “Nothing in early evolution makes sense except in the light of underlying bouts of chemical predetermination” [25].

In sum, all of these papers serve to richen the discussion of the RNA World, in all of its various forms. Although definitive confirmation of any of these ideas may require a time machine, I sense that we are at the precipice of a unified theory that accommodates a wide spectrum of RNA-related observations.
